# ZmTB1^be1^ Modulates Ear Branching via Releasing the Repression to *ZmWOX3a* in Maize

**DOI:** 10.3390/plants15162414

**Published:** 2026-08-07

**Authors:** Zheyu Yan, Yulan Wang, Anqi Sun, Jiuguang Wang, Zhiyun Qi, Shujun Li, Lanyousong Deng, Tingting Liu, Mengya Qian, Haitao Qian, Shunzhe Zhang, Hong Duan, Qianlin Xiao, Zhizhai Liu

**Affiliations:** 1 College of Agronomy and Biotechnology, Southwest University, Chongqing 400715, China; 13786041673@163.com (Z.Y.); ylwang020720@163.com (Y.W.); 18090310071@163.com (A.S.); wangjiuguang0@163.com (J.W.); 19978268651@163.com (L.D.); 13617618609@163.com (T.L.); 18064238802@163.com (M.Q.); 15870128445@163.com (H.Q.); 18836466257@163.com (S.Z.); dh760415627@126.com (H.D.); 2 Chongqing Academy of Agricultural Sciences, Chongqing 401329, China; qzy5609@163.com (Z.Q.); 15923148597@163.com (S.L.)

**Keywords:** maize (*Zea mays* L.), *ZmTB1^WT^* and *ZmTB1^be1^*, *ZmWOX3a*, ear branching, transcriptional regulating

## Abstract

The natural mutation of *ZmTB1^WT^* to *ZmTB1^be1^* was documented as the fundamental cause of ear branching in maize, while the molecular mechanism remains undiscovered. In the present study, we characterized both ZmTB1^WT^ and ZmTB1^be1^, and found that the structure of conserved domains, activation features, and subcellular localization of both proteins remained unchanged, whereas the mutation significantly decreased the transactivating power of ZmTB1^WT^ to the documented downstream target genes. Further DAP-qPCR identified significant enrichment of ZmTB1^be1^ within the promoter of *ZmWOX3a* (p*ZmWOX3a*), and the results of EMSA and Y1H indicated that ZmTB1^be1^ could directly bind to p*ZmWOX3a*. D-LUC assays showed that ZmTB1^WT^ significantly suppressed the activity of p*ZmWOX3a*, while this suppression effect was completely released by the mutation of ZmTB1^be1^. Furthermore, *ZmTB1^WT^* exhibited statistically higher expression levels than *ZmWOX3a* within the tissues of immature ear and axillary buds at V7 under WT background, while *ZmWOX3a* possessed significantly stronger expression signals than *ZmTB1^be1^* under *be1* background and *ZmTB1^CR^* under *ZmTB1^WT^*-edited backgrounds. The summary results of the present study hypothesize a novel cascade of ZmTB1^be1^-*ZmWOX3a* in regulating ear branching, shedding a new light in understanding the female inflorescence development in maize.

## 1. Introduction

Ear or female inflorescence of maize generally derives from the axillary meristem (AM) in nodes of the middle plant [1], which initiate floral primordia and ultimately produce mature kernels after fertilization and determine final kernel yield [2,3]. As a critical domestication-associated organ, maize ear functions as the primary sink tissue for kernel development [4]. During the vegetative–reproductive transition, AMs differentiate into inflorescence meristems (IMs), and the nodal IMs develop into cobs with larger diameters and more axillary rows [4,5,6]. Peripheral suppressed bracts (SBs) of IMs develop into spikelet pair meristems (SPMs). Individual SPMs generate a pair of spikelet meristems (SMs), which differentiate into two floret meristems (FMs) to form carpels, silks, and other floral structures [7]. IM proliferation and domain size control SPM initiation and final kernel yield [8,9,10].

Genetic studies revealed that multiple regulatory mechanisms were responsible for governing ear development in maize [3,4,6,11,12]. The RAMOSA (RA, i.e., *RA1*/*2*/*3*) pathway governs maize inflorescence architecture, and related mutations tend to induce the conversion of SPMs to branch meristems (BMs), then cause over-branched tassels and ears in maize [2,13,14,15]. *RA1* codes a C2H2 transcription factor (TF) that interacts with REL2 to assemble an inhibitory protein complex suppressing downstream targets [15,16]. *RA2* encodes a LOB-domain TF while the product of *RA3* acts as trehalose-6-phosphate (T6P) phosphatase, both of which positively activate *RA1* expression [14,17]. Besides the RA pathway, loss-of-function mutations in *FEA* and *Fas1* induce ectopic branching and ear fasciation in maize, and they function in the CLAVATA-WUSCHEL (CLV-WUS) feedback pathway [8,18]. Meanwhile, maize also harbors several CLV3-like peptides such as ZmFCP1, ZmCLE7, ZmCLE14, and ZmCLE1E5, whose mutations trigger ear enlargement and fasciation, and offer promising strategies for maize yield improvement [19,20].

In addition to both RA and CLV-WUS pathways, the domestication gene of *ZmTB1* (hereafter unified as *ZmTB1^WT^*) acts as another critical regulator coordinating plant architecture and ear development in maize. With the encoding product of TCP-family TF, *ZmTB1^WT^* displays approximately two-fold higher expression level in cultivated maize than in teosinte because of a transcriptional alteration induced by the Hopscotch transposon inserted in its upstream regulatory region [4]. The elevated expression of *ZmTB1^WT^* substantially represses tillering and ultimately shapes the plant architecture of cultivated maize varieties [4,21]. Meanwhile, *ZmTB1^WT^* was also detected in stamens and basal florets during the bisexual stage of female inflorescence development while the expression signals disappeared at the stage of sex determination [22]. Beyond its spatiotemporal expression pattern, ZmTB1^WT^ positively regulates the domestication gene of *Tga1* to modulate glume development [23,24]. ZmTB1^WT^ interacts with *etb1.2* to control internode length of maize ears, thereby influencing kernel density [25]. ZmTB1^WT^ could also upregulate *Tru1* and activate *Gt1*, thereby repressing axillary bud differentiation and ear development [26,27]. Additionally, *ZmTB1^WT^* attenuates T6P-mediated sugar signaling and the biosynthesis of ABA and JA to modulate the emerging and outgrowth of axillary buds in maize [28].

The WUSCHEL-related homeobox (WOX) family comprises plant TFs with a conserved homeodomain, which primarily governs stem cell homeostasis and embryonic development [29,30]. The first *WUS* gene was characterized in *Arabidopsis thaliana* [31]. A total of 15 *WOX* members have been identified across the *Arabidopsis* genome with diversified functions, while rice genome possesses at least 13 *WOX* genes functioning in the development of root, leaf, and internode via diverse mechanisms [32,33]. *AtWOX3* modulates petal development, and its mutation tends to cause missing or narrowed petals in *Arabidopsis* [34]. The major functions of documented *AtWOX*s and *OsWOX*s are involved in root development [35,36,37,38], while some paralogs from maize exhibit different behaviors. For example, *ZmWUS1/2* are highly expressed in axillary meristems, regulating branch development [39]. Furthermore, *ZmWox2a* modulates somatic embryo regeneration [40,41], whereas ZmWox3 constitutes a key module governing planar growth in leaves and ligular tissues [42].

In our previous work, a novel allele of *ZmTB1^WT^*, i.e., *ZmTB^be1^*, was documented as the target in regulating branched ears of *be1* [43], while the molecular mechanism of *ZmTB^be1^* remains unknown. In the present study, diverse characterizations and assays were performed, and ZmTB^be1^ was predicted to modulate the transcription of *ZmWox3a*, thereby participating in the regulation of branch development during maize female inflorescence formation. These findings hypothesize a novel insight of ZmTB1^be1^-*ZmWOX3a* into the regulatory mechanisms underlying female ear development and branch regression during maize domestication.

## 2. Results

### 2.1. Sequence Characterization of ZmTB1^WT^ and ZmTB1^be1^

**Figure 1:**
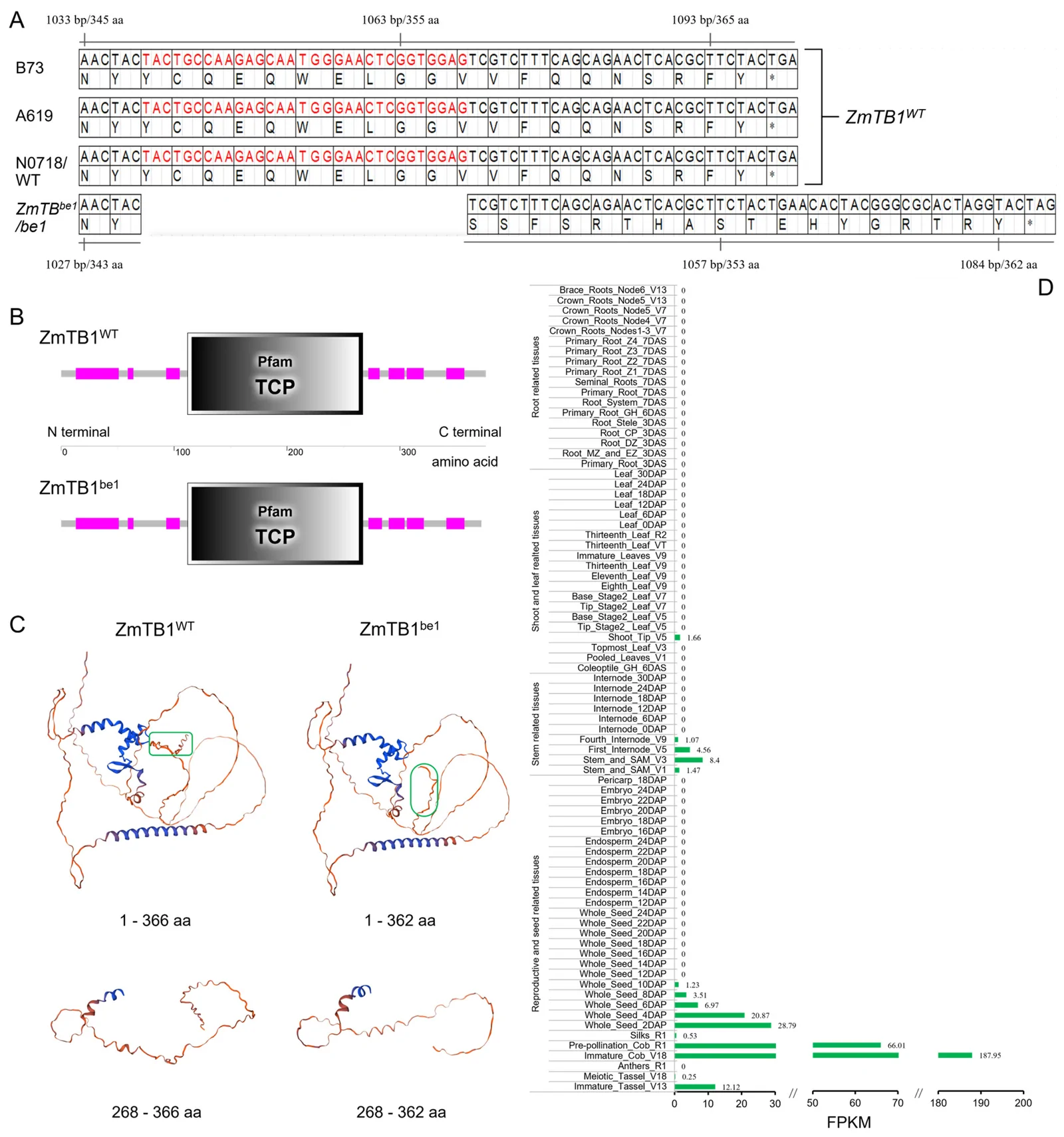
Sequence characterization of ZmTB1^WT^ and *ZmTB1^be1^*. (**A**) Schematic illustration of the 31-bp deletion within ZmTB1^be1^ (upper line) and the corresponding alterations within the coding amino acids (aa, lower line). Red letters within *ZmTB1^WT^* from B73, A619, and N0718 (WT) refer to the presence of 31-bp deletion, scales at top and bottom represent the position of coding sequence (bp) and amino acids (aa), respectively. * refers to the stop codon. (**B**) Prediction of conserved domains within the proteins of ZmTB1^WT^ and ZmTB1^be1^. Pink and black boxes refer to the conserved and TCP-family specific domains, respectively. (**C**) Tertiary structure prediction of ZmTB1^WT^ and ZmTB1^be1^. Green boxes refer to the less folded part at C-terminal of both ZmTB1^WT^ and ZmTB1^be1^. (**D**) FPKM-based expression pattern of *ZmTB1^WT^* among 79 different tissues in maize. Figures right to the green bars refer to the FPKM values that retrieved from maize genome database (www.maizegdb.org, update: 4 June 2026). DAS and DAP refer to days after seedling and days after pollination, respectively.

It was previously documented that mutation of *ZmTB1^be1^* caused a branched architecture of female inflorescence in *be1* [43]. Based on the reference genome, we retrieved the full-length sequences of *ZmTB1^WT^* from inbred lines B73, A619, and N0718 (WT), and the mutated allele of *ZmTB1^be1^* was isolated from the mutant of *be1*. Sequence alignment identified a 31-bp deletion downstream of *ZmTB1^be1^*, which causes a frameshift mutation and an additional 25 bp, generating a novel stop codon, thereby altering the C-terminal amino acid sequence of ZmTB1^be1^ (Figure 1A). Conserved domain analysis showed that the 31-bp deletion within *ZmTB1^be1^* does not disrupt the conserved domain of the encoded protein (Figure 1B). Nevertheless, tertiary structure modeling indicated changes at the C-terminal of ZmTB1^be1^, i.e., the changed amino acids resulted in less folds and loose structure (Figure 1C). Furthermore, transcriptomic datasets were retrieved from maize genome database (www.maizegdb.org) to characterize the expression pattern of *ZmTB1^WT^*. Among all 79 recorded tissues, the expression signals of *ZmTB1^WT^* were only observed within 15 tissues, among which five tissues of immature cob at V18, pre-pollination cob at R1, whole developing seeds at 2 and 4 DAP, and immature tassel exhibited relatively higher expression levels (Figure 1D).

### 2.2. Functional Characterization of ZmTB1^WT^ and ZmTB1^be1^

**Figure 2:**
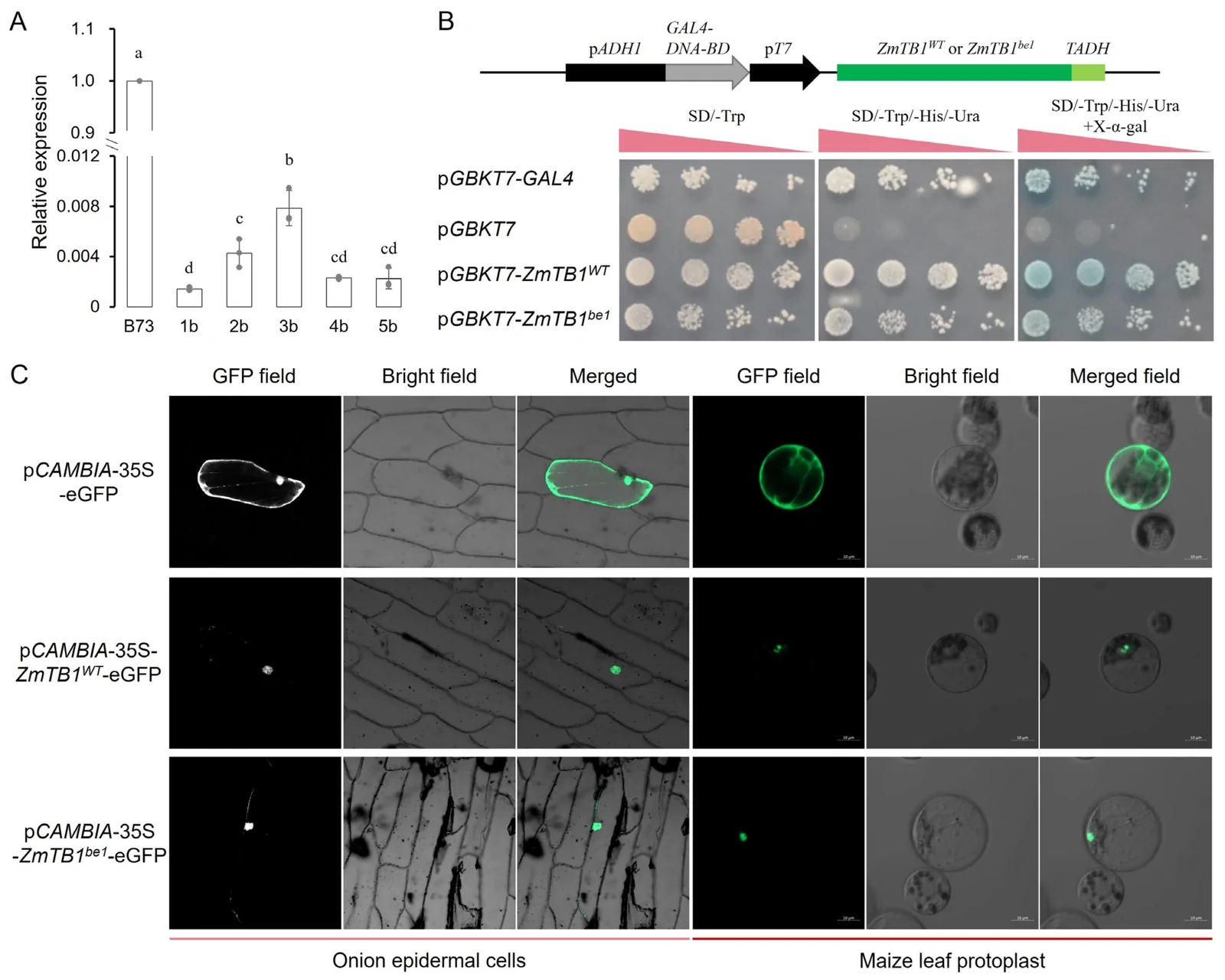
Functional Characterization of ZmTB1^WT^ and ZmTB1^be1^. (**A**) Relative expression assay of *ZmTB1^WT^* in B73 and *ZmTB1^be1^* in *be1* lines with branched ears of one (1b) to five (5b). The relative expression level of *ZmTB1^WT^* in B73 was standardized to 1, and the same letters above the columns refer to non-significance; the difference in significance level was *p* < 0.05. Multiple comparison was performed by Tukey’s HSD method via R v4.5.1 (*n* = 3). Error bars refer to standard deviation (S.D.) (**B**) Vector construction and self-activating assays of ZmTB1^WT^ and ZmTB1^be1^. (**C**) Subcellular localization of ZmTB1^WT^ and ZmTB1^be1^ within onion epidermal cells and maize leaf protoplast.

Following sequence characterization of *ZmTB1^WT^* and *ZmTB1^be1^*, the expressions of *ZmTB1^WT^* and *ZmTB1^be1^* were correspondingly profiled in B73 and *be1* lines bearing one to five branched ears (1b to 5b) (Figure 2A and Figure S1A). Compared to the relative expression level of *ZmTB1^WT^* in B73, statistically decreased expression levels of *ZmTB1^be1^* in *be1* lines were observed (Figure 2A). Notably, among *bel* lines of 1b to 3b, *ZmTB1^be1^* exhibited the highest levels within the ears of 3b, whereas the lowest expression was detected within the lines of 1b (Figure 2A). Yeast GAL4 system-based self-activating assays demonstrated that both ZmTB1^WT^ and ZmTB1^be1^ function as transcriptional activators, capable of triggering the expression of downstream reporter genes in yeast strain and exhibiting obvious self-activating activity (Figure 2B). To further explore whether the 31-bp deletion alters the subcellular distribution and functional properties of ZmTB1^be1^, we generated two fluorescent fusion constructs, *CaMV35S*::*ZmTB1^WT^-eGFP* and *CaMV35S*::*ZmTB1^be1^-eGFP*. Subcellular localization assays were subsequently performed in both onion epidermal cells and maize leaf protoplasts (Figure 2C). Fluorescence observation revealed that both ZmTB1^WT^ and ZmTB1^be1^ exclusively localize to the nucleus, indicating that the 31-bp deletion does not affect the nuclear targeting of ZmTB1^be1^ (Figure 2C).

### 2.3. Changed Transcriptional Regulation of ZmTB1^be1^ to Reported Target Genes

**Figure 3:**
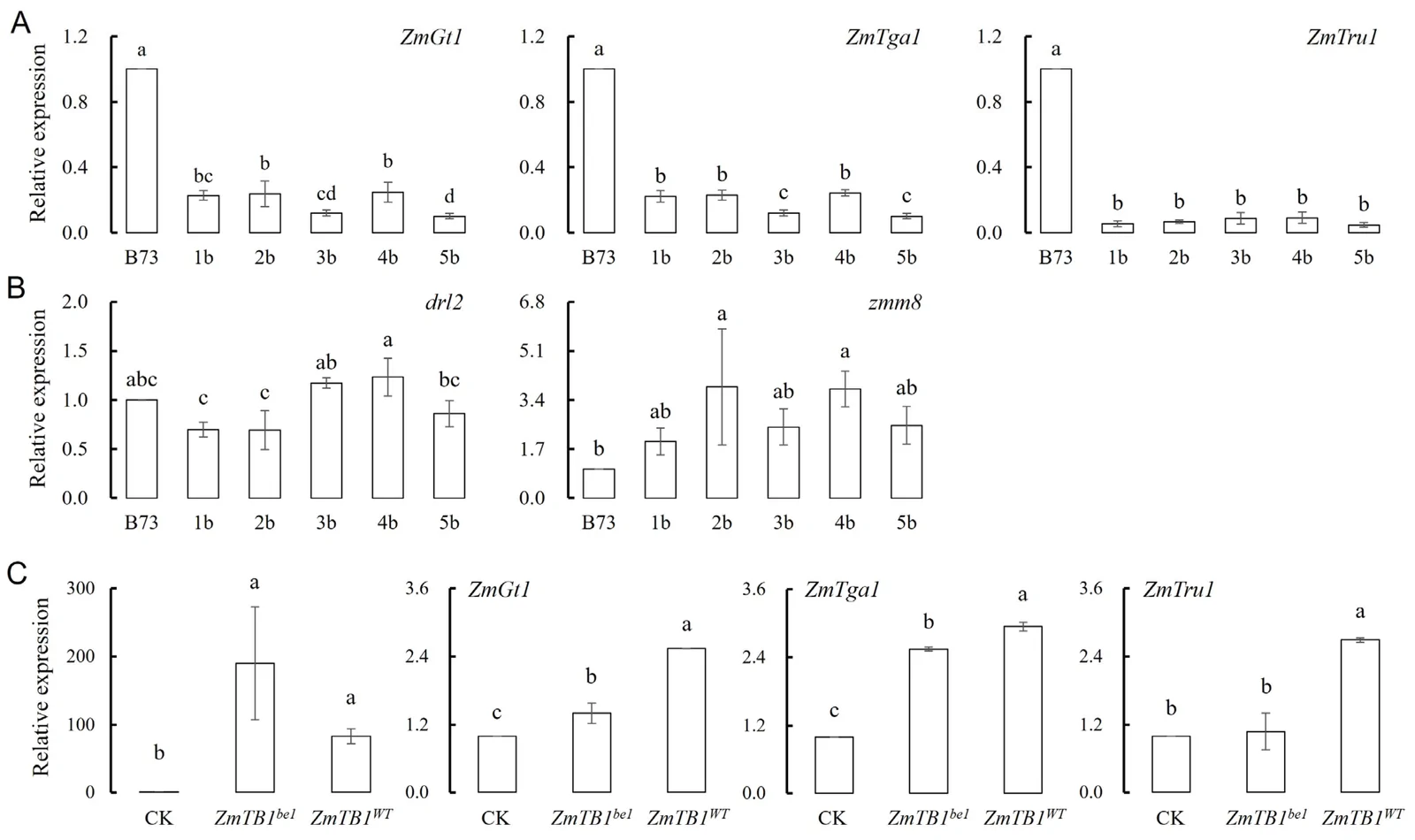
Regulatory effects of ZmTB1^WT^ and ZmTB1^be1^ on target genes. (**A**,**B**) Expression patterns of three target genes (**A**) and non-target genes (**B**) among B73 and *be1* lines. (**C**) Regulatory effects detected through transient overexpression in maize leaf protoplast. The same letters above the columns refer to non-significance; the difference in significance level was *p* < 0.05. Multiple comparison was performed by Tukey’s HSD method via R v4.5.1 (*n* = 3). Error bars refer to S.D.

ZmTB1^WT^ is documented to directly and positively modulate the expression of *ZmTga1*, *ZmTru1*, and *ZmGt1* that govern the initiation and growth of axillary buds and shank internode elongation in maize [23,24,26,27]. In this study, we further examined the transcript levels of these three documented genes within the immature ears of B73 and different *be1* lines, i.e., 1b to 5b. qRT-PCR assays revealed that the expressions of *ZmTga1*, *ZmTru1*, and *ZmGt1* changed slightly among *be1* lines of 1b to 5b, while the relative expression levels of all these three genes were significantly lower than those observed in B73, exhibiting similarly downregulated trends of ZmTB1^be1^ on these genes (Figure 3A). In addition, two non-target genes of *drl2* and *zmm8* were reported to regulate the fascicled ear performance in maize [44]. These two genes were also covered by the qRT-PCR assays, while both genes exhibited similar expression levels in B73 and different *be1* lines (Figure 3B), implying non-regulatory effects of ZmTB1^be1^ on these two genes.

To further investigate the regulation effects of downstream genes by ZmTB1^be1^, we ectopically expressed *ZmTB1^WT^* and *ZmTB1^be1^* in maize leaf protoplast. Strongly enhanced expression signals of both genes were detected after overexpression (Figure 3C). Under the overexpression background, statistically increased expression levels of both *ZmGt1* and *ZmTga1* were observed, while both genes exhibited significantly higher levels under the background of *ZmTB1^WT^* (*ZmTB1^WT^*-OE) than *ZmTB1^be1^*-OE (Figure 3C). For *ZmTru1*, similar expression trends were also detected under the transient overexpression system between *ZmTB1^WT^*-OE and *ZmTB1^be1^*-OE, though the corresponding expression level under *ZmTB1^be1^*-OE was similar to that of CK (*p* > 0.05, Figure 3C). The summary results of transient overexpression assays implied that mutation of *ZmTB1^WT^* to *ZmTB1^be1^* reduced the functioning effects on target genes.

### 2.4. ZmTB1^be1^ Regulates the Transcription of ZmWOX3a In Vitro

**Figure 4:**
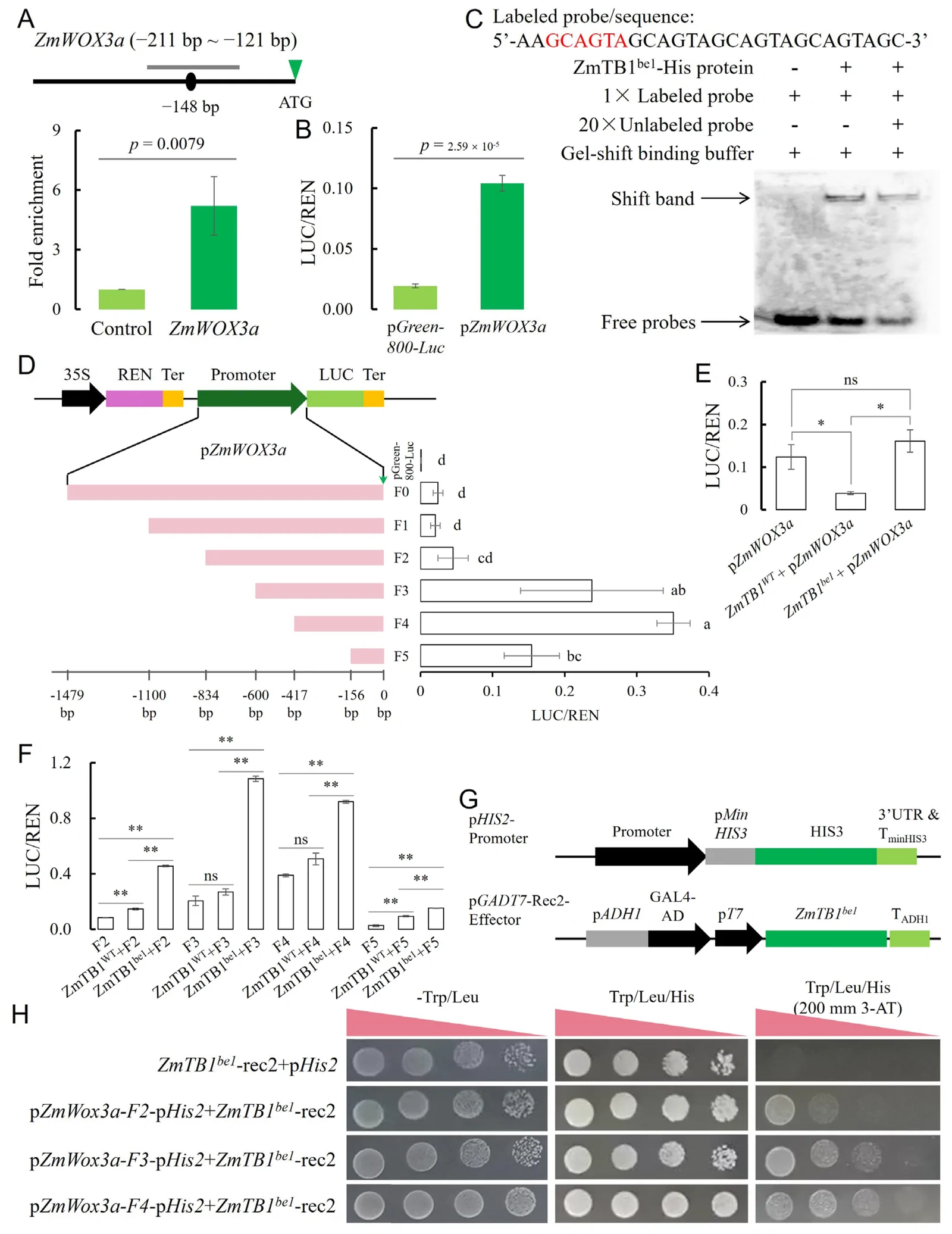
In vitro binding and effects of ZmTB1^be1^ on p*ZmWOX3a*. (**A**) The identified binding site of ZmTB1^be1^ to p*ZmWOX3a* through DAP-qPCR. (**B**) Promoter activity verification of *ZmWOX3a*. Significance between each pair of groups was identified through student’s *t*-test via R v4.5.1 (*n* = 3). (**C**) In vitro binding of ZmTB1^be1^ to p*ZmWOX3a* through EMSA. Red bases refer to the core binding motif of ZmTB1^be1^.(**D**) Activity analysis of truncated promoter fragments with different lengths of *ZmWOX3a*. The same letters at the right sides of all columns refer to non-significance; the difference in significance level was *p* < 0.05. Multiple comparison was performed by Tukey’s HSD method via R v4.5.1 (*n* = 3). (**E**,**F**) Effects of ZmTB1^WT^ and ZmTB1^be1^ on the activity of p*ZmWOX3a* with full-length (**E**) and truncated fragments (**F**). Significance between each pair of groups was identified through student’s *t*-test via R v4.5.1 (*n* = 2). * and ** referred to the significance level of *p* < 0.05 and *p* < 0.01, respectively, while ns to non-significance. (**G**,**H**) Vector construction (**G**) and binding verification of ZmTB1^be1^ to the specific fragments of p*ZmWOX3a* (**H**). Error bars in (**A**,**B**,**D**–**F**) refer to S.D.

DAP-seq profiling of our previous work identified *ZmWOX3a* as a downstream target of ZmTB1^WT^. Subsequent DAP-qPCR analysis demonstrated that ZmTB1^be1^ could directly bind to the promoter region spanning 211 bp to 121 bp upstream of ATG (−211 bp to −121 bp), the start codon of *ZmWOX3a*, with the core binding site located at −148 bp (Figure 4A). We then cloned the promoter of *ZmWOX3a* (p*ZmWOX3a*) and verified that p*ZmWOX3a* possessed the capacity to drive the expression of the LUC reporter gene (Figure 4B). A series of truncated fragments of p*ZmWOX3a* were generated, and the corresponding activities were quantified (Figure 4D). The results showed that deletion of the regions from −1479 bp to −834 bp, i.e., p*ZmWOX3a* of F0, F1, and F2, caused slight activity changes, while deletions between −834 bp to −600 bp and −417 bp to −156 bp resulted significant activity changes of p*ZmWOX3a* (Figure 4D).

In order to reveal the regulation pattern of ZmTB1^WT^ and ZmTB1^be1^ to p*ZmOWX3a*, D-LUC assays were performed under the background of maize leaf protoplast. When p*ZmWOX3a* co-transformed with *ZmTB1^WT^*, significantly decreased activity was observed in p*ZmWOX3a*, while the opposite trend was identified in co-transformation with *ZmTB1^be1^* (Figure 4E). These results suggest that ZmTB1^WT^ exhibits repression in the activity of p*ZmWOX3a*, whereas the mutation of ZmTB1^WT^ to ZmTB1^be1^ significantly weakened such repression. Similar or even enhanced trends were also detected among D-LUC assays with truncated fragments of p*ZmWOX3a*, i.e., F2 to F5 (Figure 4F). Furthermore, Y1H assays confirmed that ZmTB1^be1^ could directly interact with the truncated fragments of p*ZmWOX3a* (Figure 4G,H).

### 2.5. Transcriptional Regulation of ZmWOX3a by ZmTB1^be1^

**Figure 5:**
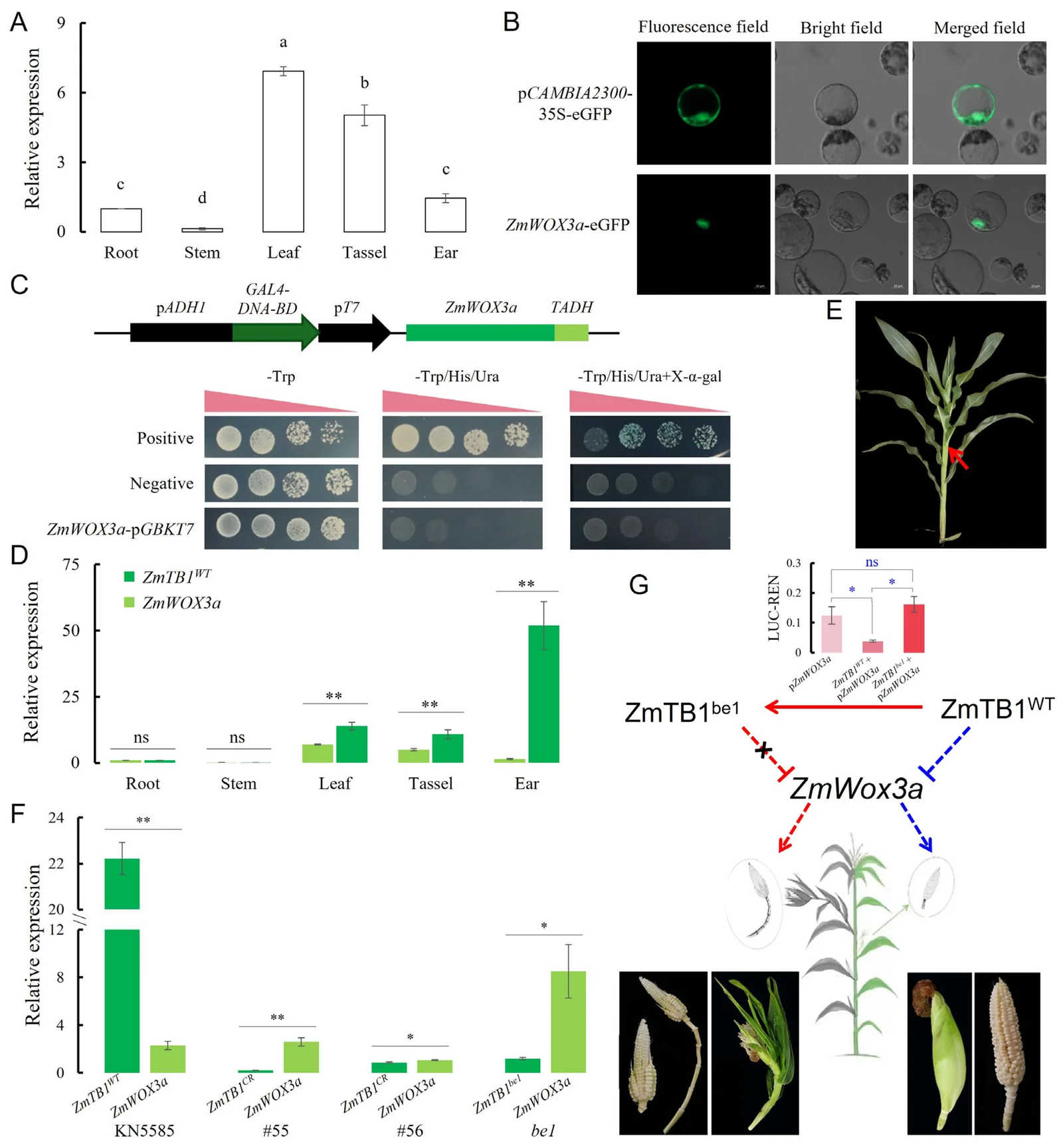
In vivo transcriptional regulation of *ZmWOX3a* by ZmTB1^be1^. (**A**) Expression pattern analysis of *ZmWOX3a*. Tissues of root, stem, leaf, tassel, and ear were sampled from the plants of B73 at the stage of V6. The same letters at the right sides of all columns refer to non-significance; the difference in significance level was *p* < 0.05. Multiple comparison was performed by Tukey’s HSD method via R v4.5.1 (*n* = 3). (**B**) Subcellular localization of ZmWOX3a in maize leaf protoplast. (**C**) Vector construction and self-activation assay of ZmWOX3a. (**D**) Relative expression of *ZmTB1^WT^* and *ZmWOX3a* among different tissues. Tissues of root, stem, leaf, tassel, and ear were sampled from the plants at the stage of V6. Significance between each pair of *ZmTB1^WT^* and *ZmWOX3a* was identified through student’s *t*-test via R v4.5.1 (*n* = 3), ns and ** referred to non-significance and significance level of *p* < 0.01. (**E**,**F**) The sampled axillary buds at V7 (**E**) and the in vivo relative expression of *ZmTB1^be1^* and *ZmWOX3a* within the buds sampled from different backgrounds (**F**). Red arrow refers to the position of sampled bud. Significance between each group was identified through student’s *t*-test via R v4.5.1 (*n* = 2), * and ** referred to the significance level of *p* < 0.05 and *p* < 0.01, respectively, while ns to non-significance. (**G**) The predicted transcriptional regulation of branched ear architecture mediated by ZmTB1^be1^-ZmWOX3a in maize. Error bars in (**A**,**D**,**F**) refer to S.D. * referred to the significance level of *p* < 0.05, while ns to non-significance.

Further characterization was performed to explore the functional properties of ZmWOX3a. Expression profiling indicated that under the reference background of B73, *ZmWOX3a* exhibited high transcript abundance in leaves and tassels, and moderate expression was also detected in root and immature ear (Figure 5A). Following protein assays revealed that ZmWOX3a was localized to the nucleus in maize leaf protoplasts without self-activating activity (Figure 5B,C).

Since ZmTB1^WT^ and ZmTB1^be1^ could directly bind to the promoter of *ZmWOX3a* in vitro, and potentially affect the following transcription (Figure 4C,E), we firstly compared the in vivo expression levels between *ZmTB1^WT^* and *ZmWOX3a* among different tissues in maize, and found that among four tissues of leaf, tassel, and immature ear, *ZmTB1^WT^* exhibited statistically higher expression levels than those of *ZmWOX3a* (*p* < 0.01, Figure 5D). Furthermore, bud samples were collected from different backgrounds of WT, *be1*, and two CRISPR/Cas9-based *ZmTB1^WT^*-edited lines of #55 and #56 at the V7 development stage (Figure 5E), and qRT-PCR assays were performed among these samples. The results showed that under the background of KN5585, WT of *ZmTB1^WT^*-edited lines, *ZmTB1^WT^* possessed a significantly higher expression level than that of *ZmWOX3a*, while the opposite trend was observed within the mutant of *be1* (Figure 5F). Within the bud samples from two *ZmTB1^WT^* edited lines, *ZmWOX3a* exhibited statistically higher expression levels than *ZmTB1^WT^*, similar to the performance observed within *be1* (Figure 5F).

Based on these assays, a summarized regulation module was hypothesized. Under the WT background, ZmTB1^WT^ generally suppresses the expression of *ZmWOX3a* to construct the normal ear architecture in maize (Figure 5G), whereas the mutation of *ZmTB1^WT^* to *ZmTB1^be1^* weakens the repression in *ZmWOX3a*, leading to the uncontrolled development of bracts, elongated shanks, and even the branched architecture of ears, like what we observed among *be1* lines (Figure 5G).

## 3. Discussion

### 3.1. ZmTB1^be1^ Weakens the Transcriptional Regulating Power of ZmTB1^WT^

Ear or female inflorescence, the major harvest and domestication related organ of maize, attracts wide and continuous attention in both labs and fields [4,21,43]. Elucidating the regulatory mechanisms underlying ear development is therefore of great theoretical significance and practical value for maize molecular breeding. Up to now, a series of key genes governing maize inflorescence development have been identified, including *ra1/2/3*, *fea2/3/4*, *Fas1* loci [8,10,13,19], and some other genes regulating kernel row number [45,46,47]. Specifically, both *ra* and *fea* related mutants display disordered inflorescence structures and irregular kernel arrangement [6,13,19]. The universal pleiotropic defects of these known mutants prevent the specific modification of female inflorescence traits, greatly limiting our understanding of the independent regulatory network underlying maize ear development and restricting the exploration of elite genetic resources for precise ear-targeted breeding.

The natural mutant of *be1* characterized in our previous study breaks the phenotypic limitation of previously reported inflorescence mutants and exhibits a unique female-specific ear branching phenotype [43]. A novel allele of *ZmTB1^WT^*, i.e., *ZmTB1^be1^*1, possessed a *be1*-specific 31 bp deletion, which was confirmed as the causal variation of branched ears in *be1* [43]. Classically, previous studies have demonstrated that *ZmTB1^WT^* acts as a key domestication gene, mainly functioning in shaping the plant architecture in maize [4,21,26,48].

In the present study, subcellular localization assays indicate that the 31-bp deletion and subsequent C-terminal frameshift mutation do not alter the fundamental subcellular localization and core transcriptional properties of both ZmTB1^WT^ and ZmTB1^be1^ (Figure 2B,C). However, further transcriptional assays revealed that mutation of ZmTB1^WT^ to ZmTB1^be1^ significantly altered the regulating power to the documented target genes (Figure 3A), implying that the C-terminal variation potentially alters the regulatory capability of ZmTB1^WT^ toward its downstream targets.

### 3.2. ZmTB1^be1^ Regulates Ear Development Through ZmWOX3a in Maize

Accumulating previous studies have demonstrated that members of the WOX family constitute a large class of plant-specific TFs that exert versatile and conserved functions in cell fate determination, organogenesis, and developmental patterning across plant species [29,30,49,50,51]. Beyond model plants, *SlWOX13* has also been verified to act as a key regulator of fruit ripening in tomato [52]. In maize, ZmWOX3a has previously been reported to regulate leaf trichome development [53]. Additionally, Kong and colleagues reported that *ZmWOX3a* could be transactivated by a ternary complex consisting of three homologous TFs of ZmSPL10/14/26, combined with auxin signaling to regulate the stigmatic papilla formation and silk development, expanding the behaviors of *ZmWOX3a* from vegetative to reproductive organs in maize [54]. A recent study in apple documented that a WOX family member of MdWUS2 could act with MdSPL9 through its N-terminal and recruit co-repressor of MdTPL through the EAR motif at its C-terminal to form transcriptional repression complex [55]. This complex tends to block the activation of MdSPL9 to *MdTCP12*/*18*, releasing the suppression to axillary buds and promoting branching in apple, implying that the collection of WOX and TCP leads to novel biological functions [55].

In the present study, we found that both ZmTB1^WT^ and ZmTB1^be1^ could directly bind to the p*ZmWOX3a*, while the opposite effects were identified in the activity of p*ZmWOX3a* (Figure 4C,E). Furthermore, *ZmWOX3a* was further detected to exhibit moderate expression levels within immature ear (Figure 5A). The mutation of *ZmTB1^WT^* to *ZmTB1^be1^* weakened the suppression to the promoter activity of *ZmWOX3a*, and *ZmTB1^WT^* exhibited statistically higher expression levels than those of *ZmWOX3a* among the same tissues of immature ear (Figure 4E and Figure 5D). Two CRISPR/Cas9-based edited lines of *ZmTB1^WT^* were selected for the in vivo verification. Under the WT background, *ZmTB1^WT^* exhibited a significantly stronger expression signal than *ZmWOX3a* within the tissue of axillary buds, while the opposite trend was observed under the mutant background containing *ZmTB1^be1^* (Figure 5F). Similar expression patterns to *be1* were also observed within the axillary buds of two *ZmTB1^WT^* edited lines (Figure 5F). It is generally believed that even within the same tissue, multiple issues, such as the amplicon properties, i.e., the amplification efficiencies of different genes/primer pairs, might lead to discrepancies in expression levels or mRNA abundances between gene pairs. However, in the present study, all assays were performed in the same sample via the same protocols with multiple biological and technical replicates. In addition, similar discrepancy trends between *ZmTB1^WT^* and *ZmWOX3a* were observed under different reference backgrounds, such as B73 and KN5586, whereas the opposite trends were observed under both *be1* and *ZmTB1^WT^*-edited backgrounds. These observations imply that the detected discrepancies between *ZmTB1^WT^*/*ZmTB1^CR^*/*ZmTB1^be1^* and *ZmWOX3a* are largely caused by the gene pairs. Collectively, these findings hypothesize a potential regulatory cascade of ZmTB1^be1^-*ZmWOX3a*, which complements the molecular network governing ear development in maize. Further genetic verifications and in vivo assays are still needed to dissect the exact molecular mechanism of this hypothesized cascade in the present study.

Interestingly, *ZmWOX3a* has been reported to be involved in the development of leaf and silk in maize, exhibiting complex functions [53,54]. For the mutant of *be1*, developed brackets and elongated shanks are closely associated with branched ears (Figure 5G) [43]. The mixed features of *be1* suggest that the hypothesized cascade of ZmTB1^be1^-*ZmWOX3a* might regulate multiple key traits of female inflorescence at the early development stage, implying diverse functions in reproductive organ development in maize.

## 4. Materials and Methods

### 4.1. Plant Materials

The plant materials utilized in this study consisted of the maize ear-branching mutant lines of *be1* that bear one to five branched ears (1b to 5b), wild-type inbred lines of N0718, the reference sequencing inbred lines B73 and A619, the gene-editing background inbred line KN5585, and two *ZmTB1^WT^*-edited lines of #55 and #56 selected from different versions of CRISPR/Cas9-based editing descendants [43]. All maize materials were cultivated at the experimental base of Southwest University in Beibei, Chongqing. The field cultivation was performed with a row spacing of 1 m and a row length of 4 m. Field management practices, including irrigation, fertilization, weeding, and pest and disease control, were performed in accordance with standard maize field cultivation protocols to ensure normal plant growth.

For indoor seedling cultivation, plump and undamaged maize seeds were selected and sown in sterilized nutrient soil. The seedlings were grown in a growth chamber at 28 °C under a 16 h light/8 h dark photoperiod for 3–5 days until germination, when seedlings with a height of 7–10 cm were transferred to continuous darkness at 28 °C for an additional 4–7 days. The etiolated second leaves were subsequently harvested for protoplast isolation and genomic DNA extraction.

### 4.2. Gene Cloning and Promoter Isolation

The coding sequence of *ZmTB1^WT^* was retrieved from the MaizeGDB database (www.maizegdb.org). Gene-specific PCR primers were designed with Primer 5, and restriction enzyme recognition sites were incorporated into the forward and reverse primers. All primers were synthesized by Sangon Biotech Co., Ltd. (Shanghai, China), diluted to a final concentration of 10 μmol/L, and stored at −20 °C.

PCR amplification was carried out in a 20 μL reaction system, and the products were resolved on 1% agarose gels, purified and extracted with a DNA purification kit, followed by ligation into the pMD19-T vector using T4 DNA ligase. Positive transformants were selected based on ampicillin resistance and verified by PCR. Sanger sequencing was performed by Sangon Biotech Co., Ltd. (Shanghai, China) to confirm the sequences of cloned gene and promoter fragments.

### 4.3. RNA Isolation and cDNA Synthesis

Total RNA was isolated from samples using the TIANGEN RNA extraction kit in accordance with the manufacturer’s protocols. RNA integrity was examined by 1% agarose gel electrophoresis, while RNA purity was determined with a Nanodrop spectrophotometer. RNA samples with the ratio of OD_260_/OD_280_ ranging from 1.8 to 2.0 were regarded as qualified. Subsequently, total RNA was reverse transcribed into cDNA following the instructions of the reverse transcription kit. The obtained cDNA was diluted to a suitable concentration and stored at −20 °C for subsequent assays.

### 4.4. Sequence Analysis and Structure Prediction

Sequence alignment was conducted using DNAMAN (Version 4.0). The amino acid composition, molecular weight, isoelectric point, and functional domains of the ZmTB1^WT^ protein were analyzed via the Sequence Manipulation Suite [56] and SMART v10 [57]. Meanwhile, the conserved domains were predicted using the Conserved Domain Database (https://www.ncbi.nlm.nih.gov/cdd, accessed on 16 April 2026), and the SWISS-MODEL server (https://swissmodel.expasy.org/, accessed on 22 July 2026) was employed for tertiary structure prediction. Considering the changes in amino acids caused by the 31 bp deletion mainly located at the C-terminal of ZmTB1^be1^, we performed a non-automatic predication segment by segment after the automatic analysis to enhance the prediction efficiency. The beginning and ending sites were artificially set for the segment covering the C-terminals of both ZmTB1^be1^ and ZmTB1^WT^ through DeepView of Project Mode [58,59].

### 4.5. Quantitative Real-Time PCR Assays

The maize *actin* gene was selected as the internal reference. Gene-specific qPCR primers were designed for the target genes *ZmTB1^WT^* and *ZmTB^be1^*, as well as the reference gene. Quantitative real-time PCR (qRT-PCR) was conducted in a 10 μL reaction system consisting of 5 μL 2 × SYBR Green qPCR Mix, 0.3 μL forward primer (10 μmol/L), 0.3 μL reverse primer (10 μmol/L), 1 μL cDNA template, and 3.4 μL sterile double-distilled water. Each sample was analyzed with two to three biological replicates (*n* = 2 to 3) and four technical replicates. The relative gene expression levels were calculated through the method of 2^−ΔΔCt^. Datasets analyses were performed through R (v4.5.1) for the significance test between sample groups.

### 4.6. Subcellular Localization

Gene-specific cloning primers for *ZmTB1^WT^* and *ZmTB1^be1^* were designed by Primer 5, with *Kpn* I and *Xba* I restriction sites engineered at their respective 5′ termini. The amplified target fragments were subcloned into the pCAMBIA2300-35S-eGFP vector to generate *35S::ZmTB1^WT^-eGFP* and *35S::ZmTB1^be1^-eGFP* fusion expression constructs. The resulting recombinant vectors were verified by colony PCR and sequencing. Maize leaf protoplasts were isolated according to Xiao et al. [60] and transformed via the PEG-Ca^2+^ method. Onion epidermal cells were prepared and transformed by a helium biolistic gun (Bio-Rad, Hercules, CA, USA) according to the protocol described by Hu and colleagues [61]. After 16 h of incubation in darkness, fluorescence signals of eGFP were visualized through fluorescence microscope LSM 800 with Airyscan (Zeiss, Jena, Germany).

### 4.7. Transcriptional Activation Activity Assay

Primer 5 was used to design cloning primers for *ZmTB1^WT^* and *ZmTB1^be1^*, with restriction sites of *Nde* I and *BamH* I incorporated at the 5′ ends. Target gene fragments were cloned into p*GBKT7* by homologous recombination to obtain *pGBKT7-ZmTB1^WT^* and *pGBKT7-ZmTB1^be1^*, and all constructs were confirmed by colony PCR and sequencing. The recombinant plasmids were transformed into Saccharomyces cerevisiae AH109 competent cells. Transformants were first selected on SD/-Trp medium and validated by colony PCR. After incubation on SD/-Trp/-His/-Ade triple dropout medium at 30 °C for 3–5 d, yeast growth was examined to determine protein auto-activation.

### 4.8. Construction of Prokaryotic Expression Vectors and Protein Purification

Cloning primers for *ZmTB1^be1^* were designed using Primer 5, with *BamH* I and *Xho* I restriction sites engineered at the 5′ end. The amplified fragment was inserted into the prokaryotic vector pET32a via homologous recombination, and the resulting construct was verified by colony PCR and Sanger sequencing.

The recombinant plasmid *ZmTB1^be1^-pET32a* was transformed into competent cells of *Escherichia coli* strain BL21. Positive transformants were cultivated in ampicillin-containing liquid LB medium at 37 °C with shaking at 200 rpm until the OD_600_ reached 0.6. Protein expression was induced by adding 0.1 mM IPTG, and the cultures were incubated at 16 °C for 12 h before bacterial cells were harvested.

Cell pellets were resuspended in PBS buffer and lysed by ultrasonication (120 W power; 5 s sonication, 10 s pause; total run time, 20 min). The sonicated mixture was subjected to centrifugation at 12,000 rpm, and the supernatant was retained for subsequent use. The recombinant protein was affinity-purified using Ni-NTA resin and eluted with PBS supplemented with 250 mM imidazole. Protein purity was assessed by SDS-PAGE. Following dialysis for desalting, the purified protein was stored at −80 °C for subsequent experiments.

### 4.9. DAP-qPCR

After our previous in vitro DNA affinity purification sequencing (DAP-seq) was performed following the protocol described by Li et al. to enrich DNA fragments bound to the ZmTB1^be1^-pET32a recombinant protein [62], primers specific to peak regions were designed based on DAP-seq-derived DNA fragments and the maize reference genome (www.maizegdb.org). qRT-PCR was used to validate the enrichment of sequences within these peaks. All primer sequences are provided in Table S1.

### 4.10. Transient Overexpression Assay

The coding sequences of *ZmTB1^WT^* and *ZmTB1^be1^* were amplified using gene-specific primers flanked with restriction sites of *BamH* I and *Sac* I as well as homologous recombination arms. The amplified products were subsequently cloned into the plant expression vector pBI221 to generate *Ubi::ZmTB1^WT^* and *Ubi::ZmTB1^be1^* plasmid (primer sequences are provided in Table S1). Maize leaf protoplasts were isolated according to Xiao et al. [62] and these overexpression constructs of *Ubi::ZmTB1^WT^* and *Ubi::ZmTB1^be1^* were introduced into maize protoplasts via the PEG-Ca^2+^ method to drive transient expression of the target genes. Empty vector *Ubi::Gus* (modified pBI221) was used as the blank control. Two independent experimental groups, i.e., overexpressing *ZmTB1^WT^* and *ZmTB1^be1^*, were included with three biological replicates for each group. Total RNA was extracted from transformed protoplasts, and qRT-PCR was performed to quantify the transcript levels of downstream genes and characterize their expression profiles. The maize *actin* gene was selected as the internal reference, while the assays and related data analyzing were the same as the description of Section 4.5.

### 4.11. Y1H Assay and EMSA

To verify the binding of ZmTB1^be1^ to the promoter of *ZmWox3a*, yeast one-hybrid (Y1H) assay was performed in this study. The yeast Y187 strain was selected as the host strain and co-transformed with recombinant vectors pHIS2-p*ZmWox3a* and pGADT7-*Rec2*-*ZmTB1^be1^*. Meanwhile, empty pHIS2 vector and pGADT7-*Rec2*-*ZmTB1^be1^* were used as negative controls. The transformed yeast cells were cultured on SD/-His/-Leu/-Trp selective medium supplemented with 200 mM 3-amino-1,2,4-triazole (3-AT) for three days. The binding interaction between ZmTB1^be1^ protein and the p*ZmWox3a* was determined by observing the growth status of yeast colonies.

In addition, electrophoretic mobility shift assay (EMSA) was conducted to biochemically confirm the direct physical interaction between ZmTB1^be1^ protein and the core *cis*-regulatory motif (5′-GCAGTA-3′) within p*ZmWox3a*. The full-length coding sequence (CDS) of *ZmTB1^be1^* was subcloned into the prokaryotic expression vector pET-32a to construct a His-tagged recombinant expression vector. Subsequently, the recombinant ZmTB1^be1^-His fusion protein was induced and purified from *E. coli* via nickel affinity chromatography. Biotin-labeled oligonucleotide probes containing four tandem repeats of the core *cis*-element of 5′-GCAGTA-3′ were synthesized and prepared for subsequent DNA-protein binding assays. The purified ZmTB1^be1^-His protein was incubated with biotinylated probes in optimized DNA-protein binding buffer. Excess unlabeled wild-type and mutant probes were added as competitive controls according to the instructions of the Chemiluminescent EMSA Kit (Beyotime, Shanghai, China). After incubation, the reaction mixtures were separated by non-denaturing polyacrylamide gel electrophoresis and electro transferred onto nylon hybridization membranes. Finally, the shifted bands of DNA-protein complexes were detected and visualized through chemiluminescence imaging.

### 4.12. Dual-Luciferase (D-LUC) Reporter Assay

The promoter regions of downstream target genes were first cloned into the pMD19 vector via primers (Table S1). Promoter fragments were re-amplified from pMD19-T templates using primers harboring restriction sites of *BamH* I and *Hind* III as well as homologous recombination sequences (Table S1). The amplified products were inserted into the pGreenII-0800-Luc vector to generate reporter constructs (pGreenII-0800-pro-Luc) via ClonExpress^®^ Ultra One Step Cloning Kit (Vazyme, Nanjing, China). Each promoter was fused upstream of the luciferase reporter to drive reporter expression for promoter activity analysis. The *Ubi::ZmTB1^WT^* and *Ubi::ZmTB1^be1^* constructs were used as effector vectors for transactivation assays.

To examine the regulatory roles of *ZmTB1^WT^* and *ZmTB1^be1^*, maize leaf protoplasts were transfected with either the reporter construct and *Ubi::Gus* as control, and co-transfected with the reporter and effector plasmids as experiment group. For each transfection, a total of 10 μg plasmids was applied at a 1:1 molar ratio of reporter to effector. Transfected protoplasts were incubated at 25 °C for 16 h prior to luciferase activity detection. Luciferase activities were measured using the Dual Luciferase Reporter Gene Assay Kit (Yeasen, Shanghai, China) according to the manufacturer’s protocols. The ratio of firefly luciferase (LUC) to Renilla luciferase (REN) activity was calculated for data normalization, which was used to quantify promoter activity and assess the transcriptional regulation exerted by *ZmTB1^WT^* and *ZmTB1^be1^*.

## 5. Conclusions

The newly documented ZmTB1^be1^ was characterized to share similar protein features to ZmTB1^WT^, while exhibiting different effects compared to the downstream target of *ZmWOX3a*. ZmTB1^WT^ was identified to significantly suppress the promoter activity of *ZmWOX3a*, whereas ZmTB1^be1^ completely released the suppression. Additionally, in axillary buds under WT background of KN5585, the expression level of *ZmTB1^WT^* was significantly higher than *ZmWOX3a*, while *ZmWOX3a* possessed more abundant transcripts in the axillary buds of *be1* and *ZmTB1^WT^*-edited lines. The integrated result of the present study implies potential association of two well-known proteins of TB1 and WUS, and hypothesizes a novel cascade of ZmTB1^be1^-*ZmWOX3a* in regulating branched ear of *be1*, shining new light for further mechanism dissection of female inflorescence development in maize.

## Data Availability

The original contributions presented in this study are included in the article/Supplementary Material. Further inquiries can be directed to the corresponding authors.

## Supplementary Materials

The following supporting information can be downloaded at: https://www.mdpi.com/article/10.3390/plants15162414/s1, Table S1: Sequence information of all primers used in the present study. Figure S1: Phenotypic and molecular illustration of *be1* and *ZmTB1^WT^*-edited lines. (A) Harvested ears bearing one to five branched ears (1b to 5b) of *be1* lines. Red # and blue * refer to the central and branched ears, respectively. (B) Molecular characterizing of *ZmTB1^WT^*-edited lines of T1 generation. “-” refers to the deleted bases, while red letter to the substituted base. (C–G) Plant architecture of WT (KN5585, C) and different *ZmTB1^WT^*-edited lines (D–G) at vegetive (V7, C–E) and reproductive (F,G) stages. (A,B,F,G) were sectioned from Ref. [43], (D,E) present #55 and #56, respectively; red arrows in (C–E) refer to the node position of sampled axillary buds.
