# Supplementary material for: ZmTB1be1 Modulates Ear Branching via Releasing the Repression to ZmWOX3a in Maize
Source: Plants (Basel). 2026 Aug 7;15(16):2414. doi: 10.3390/plants15162414 (PMC13516190; doi:10.3390/plants15162414)
Supplement: Supplementary file 1 [file plants-15-02414-s001.zip › plants-4456403-supplementary.pdf]

Table S1. Sequence information of all primers used in the present study.

Primer name	Forward sequence (5'-3')	Reverse sequence (5'-3')	Primer orientation
p*ZmWOX3a*	ACCTAAACCCTGACCGAATCG	GCCACCCTGTCCGAGTCATA	Primers for promoter cloning
p*ZmWOX3a-0800*	GTCGACGGTATCGATAAGCTTAGAGCAAGGTCGTCAGCAA	CGCTCTAGAACTAGTGGATCCGACGGCATGTCGGTCGTCGT	The primers were designed for promoter cloning and construction of pGreenII 0800 vector
p*ZmWOX3a-F1*	GTCGACGGTATCGATAAGCTTCGTTGTCCCTTTCCTCGATCTC	CGCTCTAGAACTAGTGGATCCGACGGCATGTCGGTCGTCGT	
p*ZmWOX3a-F2*	GTCGACGGTATCGATAAGCTTTCTGTCAGGCATTTGGAATC	CGCTCTAGAACTAGTGGATCCGACGGCATGTCGGTCGTCGT	
p*ZmWOX3a-F3*	GTCGACGGTATCGATAAGCTTTGCCATATTGCCATTGCCAC	CGCTCTAGAACTAGTGGATCCGACGGCATGTCGGTCGTCGT	
p*ZmWOX3a-F4*	GTCGACGGTATCGATAAGCTTTGTATAGTGTCTTCTGCATC	CGCTCTAGAACTAGTGGATCCGACGGCATGTCGGTCGTCGT	
pZmWOX3a-F5	GTCGACGGTATCGATAAGCTTTATTCACAGACAGCAGCTTC	CGCTCTAGAACTAGTGGATCCGACGGCATGTCGGTCGTCGT	
ZmTB1-UG221	CAGGTCGACTCTAGAGGATCCATGTTTCCTTTCTGTGATTCCTC	CGATCGGGGAAATTCGAGCTCTCAGTAGAAGCGTGAGTTCTGC	The primers were designed for gene cloning and construction of pBI221 vector
ZmTB^{be1}-UG221	CAGGTCGACTCTAGAGGATCCATGTTTCCTTTCTGTGATTCCTC	CGATCGGGGAAATTCGAGCTCCTAGTACCTAGTGCGCCCGTAG	
p*ZmWOX3a-pHis2-F1*	GACTCACTATAGGGCGAATTCCGTTGTCCCTTTCCTCGATCTC	CGGATCGATTCGCGAACGCGTGACGGCATGTCGGTCGTCGT	The primers were designed for promoter cloning and construction of pHis2
p*ZmWOX3a-pHis2-F2*	GACTCACTATAGGGCGAATTCTCTGTCAGGCATTTGGAATC	CGGATCGATTCGCGAACGCGTGACGGCATGTCGGTCGTCGT	
p*ZmWOX3a-pHis2-F3*	GACTCACTATAGGGCGAATTCTGCCATATTGCCATTGCCAC	CGGATCGATTCGCGAACGCGTGACGGCATGTCGGTCGTCGT	
p*ZmWOX3a-pHis2-F4*	GACTCACTATAGGGCGAATTCTGTATAGTGTCTTCTGCATC	CGGATCGATTCGCGAACGCGTGACGGCATGTCGGTCGTCGT	

ZmTB1^{be1}-REC2	GTACCAGATTACGCTCATATGATGTTTCCTTTCTGT GATTCCTC	CAGCTCGAGCTCGATGGATCCCTAGTACCTAGTGCGCCCGTAG	The primers were designed for gene cloning and construction of pGADT7-REC2 vector
ZmWOX3a	AGCCAGCCATCAAATCCTAGC	AGTAGTAATTAATGGTAACAACGGC	Primers for gene cloning
ZmTB1-BK	TCAGAGGAGGACCTGCATATG ATGTTTCCTTTCTGTGATTCCTC	CCGCTGCAGGTCGACGGATCC TCAGTAGAAGCGTGAGTTCTGC	The primers were designed for gene cloning and construction of pGBKT7 vector
ZmTB1be1-BK	TCAGAGGAGGACCTGCATATG ATGTTTCCTTTCTGTGATTCCTC	CCGCTGCAGGTCGACGGATCC CTAGTACCTAGTGCGCCCGTAG	
ZmWOX3a-BK	TCAGAGGAGGACCTGCATATGATGCCGTCGCAGC AGCAGCA	CCGCTGCAGGTCGACGGATCCTCAGGCCACGTCGTGCTGCTC	
ZmWOX3a-eGFP	ATTTGGAGAGGACAGGGTACCATGCCGTCGCAGC AGCAGCA	GGTACTAGTGTCGACTCTAGAGGCCACGTCGTGCTGCTCTTC	The primers were designed for gene cloning and construction of pCAMBIA2300-35S-EGFP vector
ZmTB1-eGFP	ATTTGGAGAGGACAGGGTACC ATGTTTCCTTTCTGTGATTCCTC	GGTACTAGTGTCGACTCTAGA GTAGAAGCGTGAGTTCTGCTGA	
ZmTB1^{be1}-eGFP	ATTTGGAGAGGACAGGGTACC ATGTTTCCTTTCTGTGATTCCTC	GGTACTAGTGTCGACTCTAGA GTACCTAGTGCGCCCGTAGTG	
actin	TGAGCGGGAGATTGTCAGGGACATA	CGATTCCATGCCAACAAGGGATGGT	These primers were used for qRT-PCR analysis.
ZmWOX3a-q	CAAGAACGTCTTCTACTGGTTCCAG	ACGGGGAGAGCAGCTGGTGCCCGTA	
ZmTB1-q	CTCATCGTTGTCCATGAACATGCCG	AAGACGACTCCACCGAGTTCCCATTG	
ZmGt1-q	CTCCGGTGGTGAGCAGAAGAAG	CATGAGCTTGCTCTTGTGGC	
ZmTga1-q	AAGCCACAGCCAGACACCA	CCCAGAGCCACGAAATGC	
ZmTru1-q	GCGCAGTTCGGCAAGATG	AGGGCCAAACGGATTATCTCAC	
ZmZmm8-q	CTCCAACTACAGCACACAGGAA	CTAATCGTTGGTGCAGTTTCAA	
ZmDrl2-q	AGCCAGATATCCCTCACAGG	GCGTACAATCCAACAAGAGC	
UBI	GACGAGTCTAACGGACACCA	ATTTCTGGATGCCGACAGCG	These primers were used for DAP-qPCR analysis
ZmWox3a-Pro DAP-seq	CTCATCTCTGCCTCTTCTGATCC	TGAGGGAAGGGAGAAGGACGCGTAC	

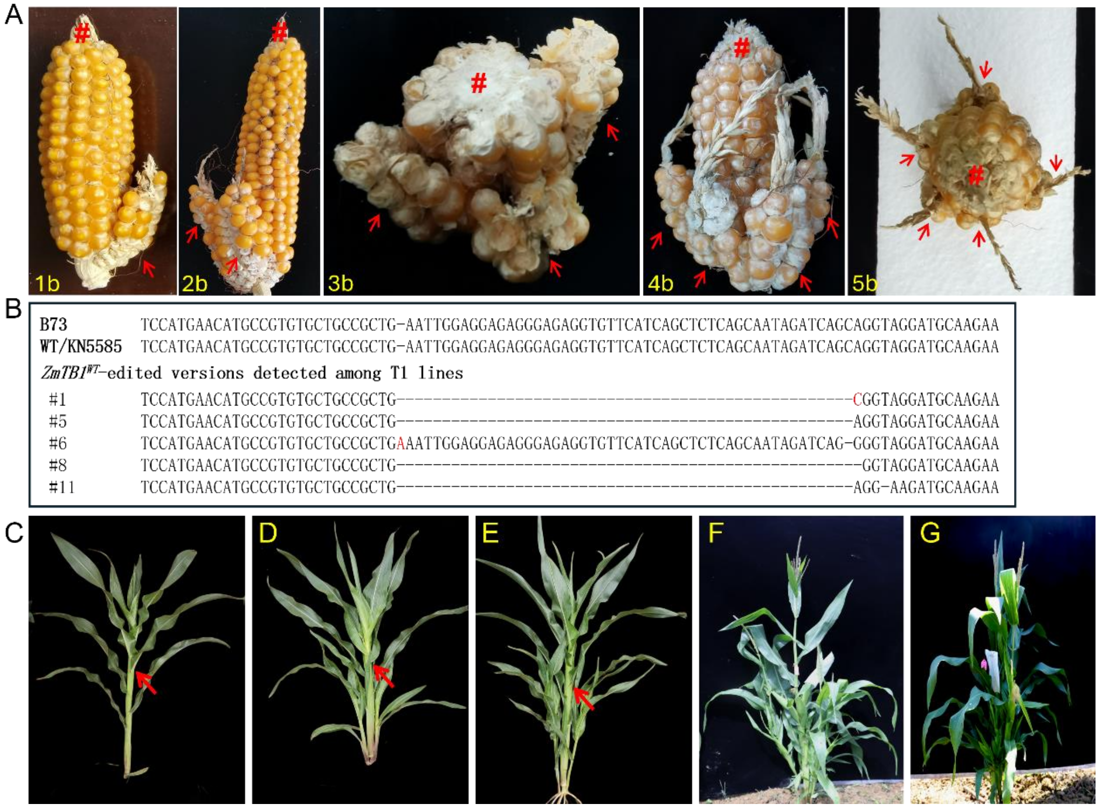

Figure S1: Phenotypic and molecular illustration of *be1* and *ZmTB1*^{WT}-edited lines. (**A**) Harvested ears bearing one to five branched ears (1b to 5b) of *be1* lines. Red # and arrows correspondingly refer to the central and branched ears. (**B**) Molecular characterizing of *ZmTB1*^{WT}-edited versions among all lines of T1 generation. "-" refers to the deleted bases, while red letters to the substituted (#1) or inserted (#6) bases. (**C-G**) Plant architecture of WT (KN5585, **C**) and different *ZmTB1*^{WT}-edited lines (**D-G**) at vegetive (V7, **C-E**) and reproductive (**F-G**) stages. D and E present #55 and #56, respectively; red arrows in C-E refer to the node position of sampled axillary buds.
